# Psychometric Validation of the Multidimensional Scale of Perceived Social Support During Pregnancy in Rural Pakistan

**DOI:** 10.3389/fpsyg.2021.601563

**Published:** 2021-06-15

**Authors:** Maria Sharif, Ahmed Zaidi, Ahmed Waqas, Abid Malik, Ashley Hagaman, Joanna Maselko, Katherine LeMasters, Rakhshanda Liaqat, Samina Bilal, Tayyaba Bibi, Ikhlaq Ahmad, Siham Sikander, Atif Rahman

**Affiliations:** ^1^Human Development Research Foundation, Islamabad, Pakistan; ^2^Department of Primary Care & Mental Health, Institute of Population Health, University of Liverpool, Liverpool, United Kingdom; ^3^Faculty of Research, Rawalpindi Medical University, Rawalpindi, Pakistan; ^4^Department of Population Health Science, Global Institute of Human Development, Shifa Tameer-e-Millat University, Islamabad, Pakistan; ^5^Department of Social and Behavioral Sciences, Yale School of Public Health, Yale University, New Haven, CT, United States; ^6^Department of Epidemiology, Gillings School of Global Public Health, University of North Carolina at Chapel Hill, Chapel Hill, NC, United States; ^7^Department of Public Health, Health Services Academy, Islamabad, Pakistan

**Keywords:** social support (MeSH term), social support, validation, psychometric, antenatal depression, postpartum depression, perinatal depression

## Abstract

**Background:** The Multidimensional Scale of Perceived Social Support (MSPSS) is a short and reliable instrument that assesses perceived social support from the social network of an individual. A previous study in Pakistan among postpartum women has demonstrated a unidimensional factor structure in contrast to the original three-factor structure. The emergence of a one-factor structure for postpartum women in Pakistan may be due to traditional postpartum practices unique to the women of the subcontinent. Building upon the previous evidence, this study aims to explore the psychometric properties of MSPSS among pregnant women in their third trimester in rural Pakistan.

**Methods:** A cross-sectional survey was conducted from October 2014 to February 2016, in rural Pakistan. A sample of 1,154 pregnant women (aged ≥ 18 years) in their third trimester who were registered with the local Lady Health Worker Program and were living in the north of the Punjab Province was included in this study. They were assessed using Urdu translated scales of Patient Health Questionnaire, MSPSS, Maternal Social Support Index, and Perceived Stress Scale. Principal Axis Factoring was used to assess the construct validity of the MSPSS.

**Results:** The MSPSS scale showed an excellent internal consistency, yielding a Cronbach's α-value of 0.933. The MSPSS scale exhibited an excellent construct validity, and confirmatory factor analysis retained three factors (family, friends, and significant others) for both the depressed and non-depressed samples. Internal reliability and construct validity were also established.

**Conclusion:** The psychometric findings suggest that the tridimensional structure of MSPSS is a valid and reliable measure of perceived social support among the Pakistani population with and without perinatal depression. The perceived social support is an important predictor of maternal mental well-being and psychopathologies, and the MSPSS can serve as a useful tool in mental health research in Pakistan.

## Introduction

In recent decades, the psychosocial aspects of pregnancy have garnered significant attention in the Indian subcontinent. Situated in this region, the country of Pakistan is home to over 210 million people, making it the sixth most populous nation in the world (UNICEF, 2018). A predominantly Muslim nation, Pakistan is an ethnically diverse country where family units are predominantly patriarchal (Waqas et al., 2020). Although pregnancy in this culture is generally seen as a time of fulfillment and joy, for many it brings a plethora of psychosocial stress, owing to the religio-cultural stigma rooted in gender discrimination and patriarchy (Waqas et al., 2015a). These sociocultural norms and psychosocial stressors are also important predictors of the mental health of women during the perinatal period (Waqas et al., 2015a).

Research on the psychosocial aspects of pregnancy, especially in the context of Indo-Pakistani culture, has shown social support to be a strong predictor of a healthy pregnancy (Waqas et al., 2015a; Naveed et al., 2018). Social support from the social network of a person spans across several constructs, including the provision of emotional, informational, and practical physical support (financial and material) during the time of need (Nakigudde et al., 2009; Ng et al., 2015; Dambi et al., 2018). Social support plays a great role in maternal mental well-being, self-confidence, and self-esteem and buffers stress and depressive symptoms (Tonsing et al., 2012; Ekbäck et al., 2013). Conversely, poor social support has been strongly associated with postnatal depression, anxiety, and maternal trauma (Grav et al., 2012; Yildiz and Aşti, 2015; Hannan et al., 2016). Poor social support also indirectly contributes to increased healthcare costs owing to its association with poor mental and physical health, poor antenatal healthcare-seeking practices, child neglect, lack of folic acid supplementation, anemia, and poor experiences during the labor procedure (Grav et al., 2012; Arnold-Baker, 2015; Biaggi et al., 2015). Recognizing this, several psychosocial interventions targeting perinatal mental health have social support as an important therapeutic element (Rahman et al., 2008; Tripathy et al., 2010).

Despite the strong relevance of social support in Pakistan and neighboring countries, research on it is limited to the context of high-income countries. Very little effort has been invested in this region in elucidating the theoretical constructs and characteristics of social support in south Asia (Heaney, 2008). Therefore, most of the theoretical underpinnings and associated knowledge have been derived and adapted from Western nations. One such challenge has been the development and cross-cultural adaptations of psychometric scales to assess social support in Pakistan (Ekbäck et al., 2013; Wang et al., 2017). All the scales assessing social support networks in Pakistan have been derived from the Western world. While these scales were not intended for use, specifically among women in the perinatal period, these were carefully translated and cross-culturally adapted for use in this population (Ekbäck et al., 2013; Wang et al., 2017). Two of these frequently utilized scales are the Multidimensional Scale of Perceived Social Support (MSPSS) (Ekbäck et al., 2013; Wang et al., 2017) and the Social Provisions Scale (SPS) (Russell and Rose, 1987). Both of these scales have previously been translated and adapted for use in Pakistan (Akhtar et al., 2010). The MSPSS has been favored over the SPS (Rizwan and Syed, 2015) in the literature on maternal depression because of its relevance to the Pakistani culture, better comprehensibility, and easy use (Tonsing et al., 2012).

The MSPSS, developed by Zimet et al., is the most frequently used instrument to assess maternal social support in Pakistan (Ekbäck et al., 2013; Wang et al., 2017). This scale was translated into Urdu, the national language of Pakistan, and validated for use among Pakistani women (Akhtar et al., 2010). It evaluates the perception of respondents of the level of support they receive from their social network (Wongpakaran, 2011). It comprises 12 items that assess social support from family, friends, and significant other (Tonsing et al., 2012; Dambi et al., 2018). Studies using factor analysis around the world favor a trifactorial structure of the MSPSS (i.e., friend, family, and significant other) (Tonsing et al., 2012; Wang et al., 2017; Laksmita et al., 2020).

It was found to be reliable, short, comprehensible, easy to administer, and psychometrically sound for use among Urdu-speaking Pakistani migrants in Hong Kong (Tonsing et al., 2012). In Hong Kong, items about social support from significant others in the Urdu version of the MSPSS were loaded onto the family subscale, thus yielding a two-factor structure (a) social support by friends and (b) wider family, instead of the original three-factor structure (Tonsing et al., 2012). It was later cross-culturally adapted for use among postpartum women in Pakistan, by Akhtar et al. (2010). However, Akhtar et al. (2010) found it to have a unidimensional construct in Pakistan, perhaps due to its relevance to postpartum women and the traditional birthing practice of *chilla* in the postpartum period (Lemasters et al., 2020). In addition, this disparity in factor structure may have resulted due to traditional statistical heuristics utilized by Akhtar et al. (2010). The authors employed a dimension reduction technique (principal component analysis) rather than factor analytic techniques to delineate latent factors in psychometric instruments. Moreover, their decision for retaining the number of factors/subscales was based on the traditional heuristics of *eigenvalue* rather than the Cattell's Scree plot or parallel analysis (Gaskin and Happell, 2014). Another study conducted among the Pakistani population, specifically non-pregnant women, tested the validity of the MSPSS in Urdu employing only confirmatory factor analysis (CFA). There was no reporting of reliability estimates or exploratory factor analysis in this study (Qadir et al., 2013; Dambi et al., 2018). Therefore, there is a paucity of adequate validation data for the MSPSS among pregnant women in Pakistan. The current study examines the psychometric properties of the Urdu translation of the MSPSS among pregnant women in Pakistan with a special emphasis on its construct validity. We hypothesize that the three-dimensional construct of the MSPSS scale yields better goodness of fit than one- or two-factor structures reported in previous literature (Akhtar et al., 2010; Tonsing et al., 2012).

## Methodology

### Study Design and Participants

A cross-sectional survey was conducted among pregnant women in their third trimester, from October 2014 to February 2016, in Tehsil Kallar Syedan, Rawalpindi district of the province of Punjab, Pakistan. Specifically, this study was embedded in the baseline assessment of the SHARE cluster randomized controlled trial (RCT) and the Bachpan cohort (Sikander et al., 2019a). This RCT was aimed at evaluating the effectiveness of peer-delivered Thinking Healthy Programme for perinatal depression in Pakistan. Further details of the study design and results pertaining to the effectiveness of the Thinking Healthy Programme can be read elsewhere (Sikander et al., 2015; Turner et al., 2016). This area was selected because of its geographical, cultural, and economic and social homogeneity. The study received ethical approvals from the Human Development Research Foundation, Pakistan and the Duke University, Durhan, North Carolina (IRB/2014/002 and Pro00047609, respectively).

Aided by a team of Lady Health Workers (LHWs), a team of female interviewers identified and approached pregnant women for recruitment in the study. All pregnant women on the registers of the government-employed community health workers (called LHWs) in the basic health units and aged 18 or above were included in the baseline survey. However, those women who reported severe medical or psychiatric illness warranting hospital admission were excluded. Written informed consent was taken from all the participants who were then ensured confidentiality and use of data for academic purposes only. The respondents were interviewed by using a pretested survey questionnaire consisting of participant demographic and socioeconomic characteristics, MSPSS, Maternal Social Support Index (MSSI), Perceived Stress Scale (PSS-10), and the Patient Health Questionnaire (PHQ-9).

### Scales for Interviews of Study Participants

In addition to the MSPSS, several other psychiatric rating scales were used in this study including the PSS-10, MSSI, and the PHQ-9. PSS-10 is used for measuring the perceived stress levels among the general population (Waqas et al., 2015b). It comprises 10 items which inquire about nervousness, stress, and ability to cope with stressors in daily life. The total score on the PSS-10 ranges from 0 to 40, where higher scores correspond to higher stress levels (Cohen, 1994). This scale is found to be valid and reliable in studies conducted around the globe.

The MSSI was used to assess objective social support levels among pregnant women rather than perceived social support, which is a focus of the MSPSS scale. The Urdu version of the MSSI has previously undergone a significant cross-cultural adaptation in the Kallar Syedan region (Sikander et al., 2015). It inquires the study participants about the qualitative and quantitative aspects of social support received in her household. It focuses on the assistance received by the study participants in completing the household tasks and childcare, satisfaction with relationships with other family members, and availability and degree of emergency help offered to her in the community (Sikander et al., 2015). In contrast to the MSPSS, it has more objective measures of social support and includes statements like, “who fixes meals?”

Screening of depression was done by using the Urdu version of the PHQ-9. This scale has previously been found to valid and reliable for use in this population (Gallis et al., 2018). It comprises nine items pertaining to depressive symptoms such as sadness, feelings of guilt, and anhedonia. It records responses on a 4-point Likert scale response system ranging from not at all (0) to always (3). It has shown excellent reliability and validity, with a sensitivity and specificity of 94.7 and 88.9%, respectively, at a cut-off score of 10 (Gallis et al., 2018).

### Statistical Analysis

For the sample size calculation of the study, *post-hoc* estimation was conducted. Our sample size of 1,154 pregnant mothers exceeded the recommendation of an *excellent* sample size of 1,000 participants as suggested by Comrey and Lee (Comrey and Lee, 1992). Data analyses were conducted using the IBM SPSS (v. 25, Armonk, NY, USA: IBM Corp) and FACTOR software (Lorenzo-Seva and Ferrando, 2006). Normality and floor and ceiling effects in MSPSS scores among participants were assessed by visualizing histograms. Floor and ceiling effects were considered significant when ≥20% of the respondents scored either the lowest or highest possible score on the MSPSS.

Thereafter, the items of the MSPSS scores were subjected to the reliability analysis using the SPSS software. The reliability of the overall scale and individual items was judged to be adequate at Cronbach's α ≥ 0.70. The convergent validity of the scale was assessed with inter-item correlations, considered adequate at 0.20. Thereafter, the item-level data of the MSPSS scores were subjected to exploratory principle axis factoring (PAF) analysis with oblique rotation (identifies simple structure pattern). This method was favored over other methods of factor analysis such as the maximum likelihood method and the least squares method because it is fairly robust to the assumption of multivariate normality (Gaskin and Happell, 2014). PAF was used to ascertain the factor validity for MSPSS, because it is robust to assumptions of multivariate normality. Adequacy of the study sample for the factor analysis was assessed using the Kaiser–Meyer–Olkin (KMO) measure of sampling adequacy and Bartlett's test of sphericity. The number of factors to retain was assessed using four criteria including the Cattell's Scree plot, Horn's parallel analysis, Velicer's MAP test, and Hull's method. During the PFA analysis, only those items that had a communality ≥0.20, a KMO value obtained in the anti-image matrix ≥0.60, and a factor loading ≥0.30 were considered fit to be included in the scale.

Lastly, the factor structure obtained in the exploratory analyses was tested for its goodness of fit using the CFA. These analyses were conducted using the FACTOR software (v. 10) and AMOS (v. 25, AMOS Development Corp., Wexford, PA, USA) (Lorenzo-Seva and Ferrando, 2006). CFA was conducted with the unweighted least squares method using Pearson's correlation matrix. The factor structure of the MSPSS was found to have acceptable goodness of fit at a cut-off value of 0.90 for several indices of goodness of fit, including the comparative fit index (CFI), normed fit index (NFI), incremental fit index (IFI), goodness-of-fit index (GFI), and adjusted goodness-of-fit index (AGFI). Root mean square of residuals (RMSR) was considered acceptable if it was not significantly greater than Kelley's criterion. Both the EFA and CFA were performed after splitting the dataset randomly into two parts comprising 70 and 30% of the sample, respectively.

Divergent validity of MSPSS was assessed by correlating it with scores on PHQ-9 and PSS-10, where a negative correlation of MSPSS with PHQ-9 and PSS-10 corresponded to an acceptable divergent validity. The concurrent validity of MSPSS was assessed by correlating it with MSSI scores, with a positive correlation between them being considered acceptable. All analyses were run separately for the participants with or without depressive symptoms to ascertain the construct validity of MSPSS in the two groups.

## Results

### Characteristics of Participants

There were a total of 1,154 mothers, including 570 mothers with depression (PHQ-9 ≥ 10) and 584 without depression (PHQ-9 <10). The mean age of the participants was 26.7 (4.6) years. The mean education level of the participants was 7.7 years (4.5). Most of the participants reported having a joint family structure (65.8%), followed by nuclear (22.5%) and multiple households (11.7%) (Table 1). Mean score on the MSPSS scale was 39.7 (10.5), on the family subscale was 14.1 (4.2), on the subscale of friends was 11.3 (4.6), and on the significant other subscale was 14.2 (4.1).

**Table 1:** Characteristics of all the pregnant women of Bachpan cohort (*N* = 1,154).

	**All women**	**Depressed women**	**Non-depressed women**
	**(*N* = 1,154)**	**(*N* = 570)**	**(*N* = 584)**
**Characteristic**	**Mean (SD) or %**	**Mean (SD) or %**	**Mean (SD) or %**
Age at baseline	26.69 (4.55)	27.03 (4.79)	26.37 (4.27)
Maternal education	7.7 (4.47)	6.81 (4.43)	8.56 (4.35)
Paternal education	8.62 (3.42)	8.09 (3.49)	9.15 (3.27)
SES asset index score (baseline)	−0.0036 (1.63)	−0.44 (1.71)	0.42 (1.42)
Number of children (baseline)	*N* = 881 (1.93) (1.33)	*N* = 468 (2.1) (1.41)	*N* = 413 (1.74) (1.2)
**Family structure**			
Nuclear	260 (22.5%)	142 (24.91%)	118 (20.21%)
Joint/extended	759 (65.77%)	346 (60.7%)	413 (70.72%)
Multiple households	135 (11.7%)	82 (14.34%)	53 (9.08%)

### Face Validity

The translation and adaptation process of the MSPSS has been described in detail in previous publications (Akhtar et al., 2010; Tonsing et al., 2012). To ensure good fidelity, the cultural adaptation process of the MSPSS was done in two phases. First, following a standardized procedure, key informant interviews and focus group discussions with pregnant women were conducted to develop a cultural understanding of the difficult concepts embedded in the MSPSS. Thereafter, a committee of experts in psychology and psychiatry convened the translation process of MSPSS according to the MAPI guidelines. The Urdu translation of the MSPSS is provided in Supplementary File 1.

### Floor and Ceiling

**Figure 1:**
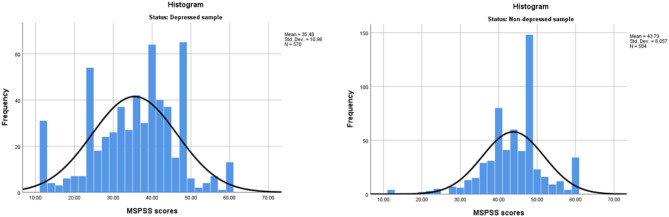
Histograms presenting distribution of the multidimensional scale of perceived social support (MSPSS) scores among women with depression and healthy women.

No floor and ceiling effects were observed among either of the study samples (Figure 1). Among patients with depression, 5.4% of the participants scored the lowest possible scores of 1, and only 2.1% scored the maximum possible score of 5. Among non-depressed participants, only 0.7% scored the lowest possible score and 5.1% scored the highest on MSPSS.

### Reliability Analysis

The MSPSS scale showed an excellent internal consistency, yielding a Cronbach's α*-*value of 0.93. The sensitivity analysis revealed no significant changes in the Cronbach's α-value when individual items were removed. The Cronbach's α remained > 0.92 in the sensitivity analyses. The corrected item-total correlations remained > 0.20, with a minimum correlation value of 0.64 for item 10 *(I can count on my friends when things go wrong*) and a maximum correlation value of 0.78 for item 8 (*My family is willing to help me make decisions*).

Reliability analyses were also run for the individual subscales of the MSPSS Table 2. The subgroup assessing social support from family also revealed an excellent internal consistency of the subscale. The Cronbach's alpha for this subscale was 0.95 with no significant changes with the removal of individual items from the subscale. The corrected item-total correlation ranged from 0.88 to 0.92. The subscale assessing social support from friends also revealed a slightly excessive Cronbach's alpha value of 0.96. The subscale assessing social support from significant others revealed a Cronbach's alpha value of 0.92. The removal of item 2 and item 4 from the scale lowered the alpha value to 0.88 and 0.89, respectively. The corrected total-item correlation for each of the items ranged from 0.84 to 0.77. The corrected item correlation for this scale ranged from 0.89 to 0.92, and the Cronbach's α item did not reveal any significant changes with the deletion of individual items from the subscale.

**Table 2:** Reliability analysis exhibiting internal consistency of MSPSS for assessing social support during pregnancy in rural Pakistan.

**Item**	**All women (0.93)**			**Non-depressed women (0.90)**			**Depressed women (0.93)**		
	**Item-total correlation**	**Alpha if item deleted**	**Mean (SD)**	**Item-total correlation**	**Alpha if item deleted**	**Mean (SD)**	**Item-total correlation**	**Alpha if item deleted**	**Mean (SD)**
There is a special person who is around when I am in need	0.671	0.928	3.516 (1.17)	0.507	0.895	3.93 (0.92)	0.683	0.927	3.098 (1.26)
There is a special person with whom I can share my joys and sorrows	0.721	0.926	3.562 (1.13)	0.588	0.892	3.92 (0.89)	0.736	0.925	3.198 (1.24)
I have a special person who is a real source of comfort to me	0.710	0.926	3.629 (1.10)	0.577	0.892	3.97 (0.84)	0.723	0.925	3.28 (1.21)
There is a special person in my life who cares about my feelings	0.700	0.927	3.54 (1.14)	0.555	0.893	3.90 (0.90)	0.716	0.926	3.16 (1.24)
My family really tries to help me	0.751	0.925	3.53 (1.09)	0.659	0.889	3.90 (0.81)	0.743	0.924	3.15 (1.21)
I get the emotional help and support I need from my family	0.766	0.924	3.55 (1.10)	0.678	0.888	3.932 (0.80)	0.756	0.924	3.16 (1.21)
I can talk about my problems with my family	0.765	0.924	3.51 (1.11)	0.666	0.889	3.93 (0.81)	0.755	0.924	3.08 (1.21)
My family is willing to help me make decisions	0.771	0.924	3.54 (1.11)	0.665	0.889	3.94 (0.81)	0.770	0.923	3.13 (1.22)
My friends really try to help me	0.654	0.928	2.77 (1.20)	0.643	0.889	3.05 (1.18)	0.639	0.928	2.49 (1.15)
I can count on my friends when things go wrong	0.643	0.929	2.76 (1.20)	0.647	0.889	3.05 (1.20)	0.609	0.929	2.46 (1.13)
I have friends with whom I can share my joys and sorrows	0.663	0.928	2.86 (1.22)	0.672	0.888	3.10 (1.19)	0.648	0.928	2.62 (1.20)
I can talk about my problems with my friends	0.654	0.929	2.92 (1.22)	0.640	0.890	3.18 (1.19)	0.646	0.928	2.66 (1.21)

### Exploratory Factor Analysis

**Figure 2:**
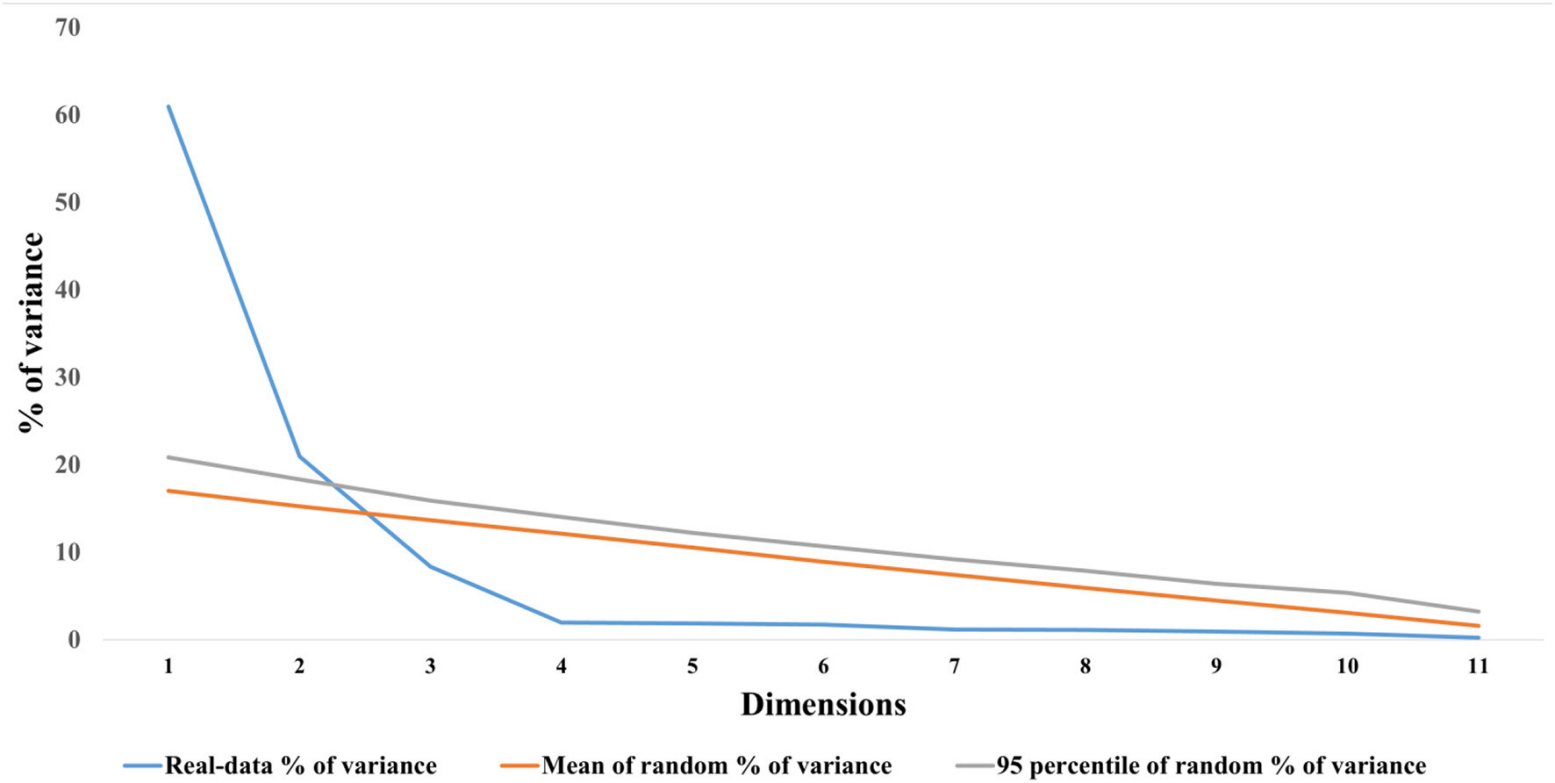
Visualization of parallel analyses results exhibiting number of factors to retain.

For the non-depressed sample, the sampling adequacy was shown to be excellent with a KMO measure of sampling adequacy of 0.89 and a statistically significant Bartlett's test of sphericity (*p* < 0.001). The anti-image correlation matrix showed that each item had a KMO value >0.50, with the lowest for item 11 (0.84) and highest for item 4 (0.93). All of the items had communalities >0.20 with the highest reported for item 11 (0.91) and the lowest for item 1 (0.50). Factors to retain were judged on both the traditional and more recent criteria: eigenvalue >1, the Scree plot, and Horn's parallel analysis. All these criteria suggested three factors to retain for the MSPSS. The cumulative variance explained by these three factors was 80.05%. The eigenvalue for the first factor was 5.74, for the second factor was 2.60, and for the third factor was 1.26 (Supplementary Figure 2), whereas the Horn's parallel analysis (Figure 2) suggested two factors to retain in contrast to eigenvalues and Scree plot criteria. All the items were strongly loaded on their respective factors. The highest factor loading was reported for item 11 (0.95) and the lowest factor for item 4 (0.61). Items 5 and 6, although belonging to the family subscale, also cross-loaded on the significant other subscale (loading > 0.30). However, the cross-loadings were minimally acceptable and were retained on their original subscale because of strong factor loading values (Table 3).

**Table 3:** Factor analysis for multidimensional scale of perceived social support (MSPSS) scale for assessing social support among pregnant women.

**Statement**	**All women**			**Depressed women**			**Non-depressed women**		
	**Friends**	**Family**	**Significant other**	**Friends**	**Family**	**Significant other**	**Friends**	**Family**	**Significant other**
There is a special person who is around when I am in need			0.779			0.709			0.772
There is a special person with whom I can share my joys and sorrows			0.954			0.854			0.977
I have a special person who is a real source of comfort to me			0.909			0.856			0.897
There is a special person in my life who cares about my feelings			0.719			0.609			0.749
My family really tries to help me		0.905			0.760			0.944	
I get the emotional help and support I need from my family		0.974			0.938			0.963	
I can talk about my problems with my family		0.857			0.842			0.848	
My family is willing to help me make decisions		0.908			0.910			0.887	
My friends really try to help me	0.892			0.872			0.906		
I can count on my friends when things go wrong	0.924			0.916			0.936		
I have friends with whom I can share my joys and sorrows	0.952			0.951			0.935		
I can talk about my problems with my friends	0.913			0.931			0.886		

For the depressed sample, similar trends were observed. The KMO measure of sampling adequacy was found to be adequate (0.90), with a statistically significant Bartlett's test of sphericity. The anti-image of the correlation matrix showed all items having a KMO value >0.50 with the highest reported for item 1 (0.95) and the lowest for item 10 (0.86). All the communality values were >0.2. The highest communality value of 0.90 was reported for item 6 and the lowest for item 1 (0.66). Three factors were retained based on the aforementioned criteria explaining 86.08% of the variance in MSPSS. Factor loadings were strong for all the items ranging from 0.75 for item 4 to 0.94 for item 11. Items 4, 6, 7, and 8 weakly cross-loaded on two scales; however, they were retained in their respective subscales because they had strong cross-loadings.

### Confirmatory Factor Analysis

**Figure 3:**
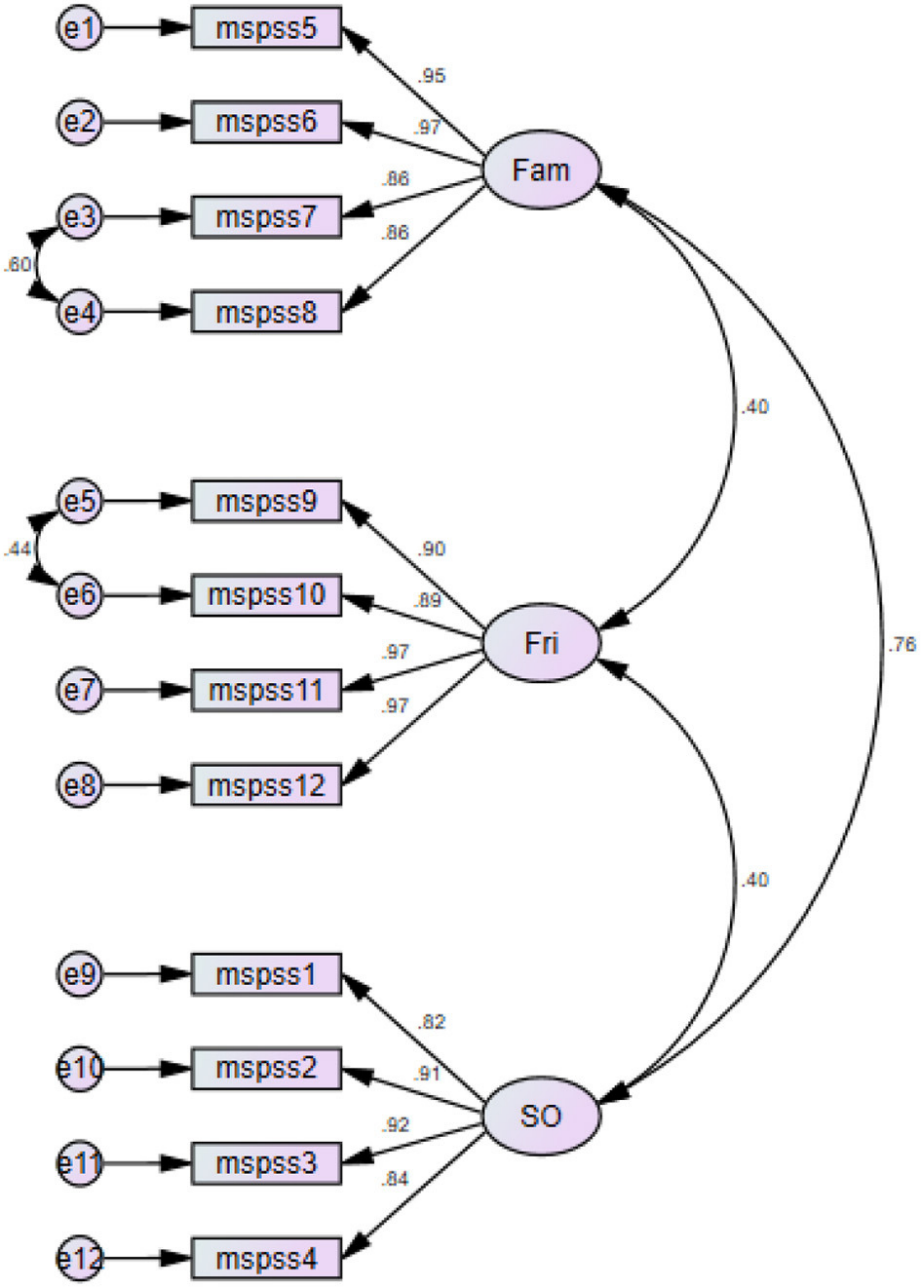
Path diagram for three-factor structure of the MSPSS scale.

Several indices were assessed to confirm the goodness of fit for a three-model structure of MSPSS (Table 4, Figure 3). The CFI was revealed to be 96%, and GFI, AGFI, GFI without diagonal values, and AGFI without diagonal values yielded indices approaching 99–100%. RMSR was estimated at 0.02, which was significantly lower than Kelley's criterion of 0.06. CFA was also run for a two-factor structure of MSPSS. This factor structure revealed acceptable goodness of fit. It yielded a CFI value of 83%, GFI of 99%, AGFI of 98%, GFI without diagonal values of 99%, and AGFI without diagonal values of 98%. RMSR was 0.06, which was not less than Kelley's criterion of 0.06. Compared with the three-factor structure, the unidimensional structure of the MSPSS proposed by Akhtar et al. (2010) revealed poorer goodness of fit in the present sample. It yielded a CFI value of 49%, GFI of 91%, AGFI of 89%, GFI without diagonal values of 89%, and AGFI without diagonal values of 86%. RMSR yielded a value (0.19) that was significantly larger than the expected mean value of RMSR calculated using Kelley's criterion (0.03).

**Table 4:** Goodness of fit indices for different factor solutions of the MSPSS.

**Factor**	**Unidimensional**	**Two-factor**	**Three-factor**
**solution**	**structure**	**structure**	**structure**
Comparative fit index	49%	83%	96%
Goodness of fit index	91%	99%	100%
Adjusted goodness of fit index	89%	98%	99%
Goodness of fit index without diagonal values	89%	99%	99%
Adjusted goodness of fit index without diagonal values	86%	98%	99%
Root mean square of residuals	0.19	0.06	0.02

### Convergent and Divergent Validity and Known Group Analysis

A good convergent validity was obtained during inter-item and item-total correlations. The corrected item-total correlations remained > 0.20, with a minimum correlation value of 0.64 for item 10 and a maximum correlation value of 0.78 for item 8. All the inter-item correlations were > 0.20, with the lowest between items 4 and 10 (0.32) and the largest between items 5 and 6 (0.90). The divergent validity of the MSPSS was found to be adequate by correlating the MSPSS scores with the PHQ-9 scores and PSS-10 scores (Table 5). The Pearson's correlation demonstrated weak-to-moderate correlations between the PHQ-9 scores and total MSPSS scores (*r* = −0.40), significant others subscale (*r* = −0.37), family subscale (*r* = −0.38), and the subscale of friends (*r* = −0.24). It also yielded a negative correlation with the PSS-10 scale (*r* = −0.27, *p* < 0.001).

**Table 5:** Mean differences on MSPSS according to status of depression.

**Subscales of MSPSS**	***t*-value**	**df**	***p*-value**	**Mean difference**	**SE difference**
Significant others	13.290	995.345	<0.001	2.98238	0.22441
Family	13.964	969.229	<0.001	3.17407	0.22730
Friends	8.204	1151.831	<0.001	2.15399	0.26254

### Concurrent Validity

The concurrent validity of the MSPSS scale was assessed by correlating it with the MSSI scale. It yielded a statistically significant, but weak, correlation with the MSPSS (*r* = 0.13, *p* < 0.001).

## Discussion

The present study revealed a tridimensional structure of the MSPSS to be valid and to be reliable for use among pregnant women in Pakistan. The three-factor structure of the MSPSS was found to have better goodness of fit than a unidimensional or a bidimensional factor structure evident in a previous literature. We found the three-factor structure to be both valid and reliable for use among Pakistani women with or without antenatal depression.

The tridimensional MSPSS was found to have excellent validity and reliability in the present study. These results are corroborated by the MSPSS scale development study (Ekbäck et al., 2013; Wang et al., 2017). Zimet et al., in this study, reported that perceived social support was obtained from three different sources: friends, family, and significant other. This contrasts with the study of Akhtar et al., who reported a unidimensional factor structure of MSPSS to be valid for use among Urdu-speaking women in Pakistan (Akhtar et al., 2010). However, this study had several limitations, for instance, a limited sample size (*n* = 325), use of dimension reduction technique (principal component analysis) rather than factor analytic techniques, and lack of CFA. Our results, however, are in agreement with that of the study by Qadir et al., who demonstrated a tridimensional factor structure to be valid; however, their analysis was restricted to CFA only with no reporting of EFA or reliability analysis.

The reliability and validity of the Urdu version of the MSPSS were reported by Akhtar et al. (2010) in Pakistan. In our study, the tridimensional factor structure conceptualizes social support from three distinct sources: family, friends, and significant other. This contrasts with the study of Akhtar et al., who reported the factor structure of MSPSS as a unidimensional construct where the family, significant other, and friends, apparently constituted the social support network of new mothers but not as distinct entities. This disparity with our findings is grounded in several rationales. Namely, the traditional heuristics employed by Akhtar et al., including relatively small sample size, use of dimension reduction techniques instead of factor analysis, and reliance on eigenvalue >1 criterion for the number of factors to retain instead of parallel analyses, may explain differences between our findings.

Factor analysis of the MSPSS revealed that there were some cross-loadings on several items of subscales of family and significant other. These cross-loadings were weak (<0.35), and therefore, we retained them in their original subscales where they demonstrated strong factor loadings (>0.75). We hypothesized that this issue may have arisen from poor translation of the term “significant other.” The term significant other should be translated as “husband or wife,” which was previously translated as “trustworthy individual.” As per the cultural norms, pre-marital relationships are discouraged in society and may be taken as negative by the study participants. In addition, our analysis revealed only a weak association between the MSPSS and MSSI scores. This is due to the heterogeneous nature of questions asked in these two scales, where the former focuses on *perceptions* and *subjective feelings*, whereas the latter leverages objective instrumental material support.

However, the most plausible rationale for this difference in our results is different study samples that assume a different social role in the Indo-Pakistani culture. The present study was conducted among prenatal women, whereas the findings of Akhtar et al. were based on postpartum women. The transition of a pregnant woman into early motherhood and the postpartum period is coupled with a stark difference in the social role in Pakistani society (Qureshi et al., 2016). This transitioning social role is also evident in Western societies, but perhaps not as much as in the Indo-Pakistani culture. After the birth of a baby, the new mothers and their families, specifically in the Punjab region, celebrate their motherhood. The postpartum woman undergoes complete bed rest, assisted by their spouses and other women in the family. They are provided with special care, energy-dense diets, porridge, and fat-dense diet. Close family members, especially their mothers, sisters, or in-laws, assume the role of their caretaker. This transitioning phase is called *chilla* in the regional language and lasts for 6 weeks after the birth of the baby. Perhaps this transition of a socially active pregnant woman to a narrow social role of a postpartum woman may explain why the family, spouses, and friends are no longer seen as three distinct sources of social support in the postpartum period (Lemasters et al., 2020).

### Implications of the Study

This tool can be used to assess social support among Pakistani mothers and a general indicator of well-being (Heaney and Israel, 2008). This scale would also prove to be valuable in assessing the effectiveness of social support interventions being tested in Pakistan. Moreover, it could also be added as an important outcome in trials of psychological therapies that leverage the concept of eliciting social support. For instance, it was an important component in the cognitive behavioral therapy-based Thinking Healthy Programme being delivered by LHWs and community health workers (Turner et al., 2016; Fuhr et al., 2019; Sikander et al., 2019b). For interventions aiming to improve social networks, the MSPSS can be used to design programs that are tailored specifically to its clients (Heaney and Israel, 2008). It can also be used in epidemiological studies that aim to study risk factors for various physical and mental illnesses, health behaviors, and assessment of capacity-building exercises of delivery agents of interventions (Heaney and Israel, 2008).

### Strengths and Limitations

This study has several strengths, including a large sample size and inclusion of both depressed and non-depressed mothers. The authors also strived to assess several aspects of validity and reliability in contrast to previous studies. However, in addition to its strengths, there are several limitations. The results of this study are specific to women in their third trimester and are not generalizable to other populations in Pakistan. Validation of the MSPSS tool should be done before testing it in populations other than pregnant women in their third trimester.

The present investigation utilizes the Classical Test Theory and presents the evidence for the factor structure of the MSPSS (Urdu). We also provide the goodness of fit for the previously reported factor structures of the Urdu version of the MSPSS. However, future research should explore the psychometric properties of the MSPSS using the item response theory (IRT), which can provide additional important information such as the item difficulty and discriminative ability (Ye et al., 2018a,b; Ye et al., 2019).

## Data Availability Statement

The raw data supporting the conclusions of this article will be made available by the authors, without undue reservation.

## Ethics Statement

The studies involving human participants were reviewed and approved by Ethical approval was taken from the ethical review committees at the University of Liverpool, United Kingdom and the Human Development Research Foundation, Rawalpindi, Pakistan, National Institute of Mental Health (NIMH) and the Institutional review board of Duke University. The patients/participants provided their written informed consent to participate in this study.

## Author Contributions

MS, AZ, AM, and AW conceptualized the study. AW performed the analyses, interpreted the results, and edited the manuscript. MS, AZ, and AM wrote the initial draft of the manuscript. SS, AR, and JM provided scientific supervision. IA, RL, SB, and TB interviewed study participants, collected the data, and performed initial analyses. All authors critically reviewed the manuscript and approved the final version for submission.

## Conflict of Interest

The authors declare that the research was conducted in the absence of any commercial or financial relationships that could be construed as a potential conflict of interest.
