# Supplementary material for: Psychometric Validation of the Multidimensional Scale of Perceived Social Support During Pregnancy in Rural Pakistan
Source: Front Psychol. 2021 Jun 15;12:601563. doi: 10.3389/fpsyg.2021.601563 (PMC8239233; doi:10.3389/fpsyg.2021.601563)
Supplement: Supplementary file 1 [file Table_1.DOCX]

Supplementary table 1: PARALLEL ANALYSIS (PA) BASED ON MINIMUM RANK FACTOR ANALYSIS

(Timmerman & Lorenzo-Seva, 2011)

**Implementation details:**

Correlation matrices analysed: Pearson correlation matrices

Number of random correlation matrices: 500

Method to obtain random correlation matrices: Permutation of the raw data (Buja & Eyuboglu,1992)

| Dimensions | Variable Real-data % of variance | Mean of random % of variance | 95 percentile of random% of variance |
| --- | --- | --- | --- |
| 1 | 61.2* | 16.9 | 20.5 |
| 2 | 21.0* | 15.2 | 18.4 |
| 3 | 8.3 | 13.6 | 15.7 |
| 4 | 2.5 | 12.0 | 13.9 |
| 5 | 1.9 | 10.5 | 12.2 |
| 6 | 1.7 | 9.0 | 10.6 |
| 7 | 1.0 | 7.5 | 9.4 |
| 8 | 0.9 | 6.1 | 8.2 |
| 9 | 0.8 | 4.5 | 6.5 |
| 10 | 0.7 | 3.1 | 5.1 |
| 11 | 0.2 | 1.6 | 3.4 |
| 12 | 0.0 | 0.0 | 0.0 |

* Advised number of dimensions: 2

Supplementary table 2: MINIMUM AVERAGE PARTIAL TEST (MAP) for number of factors to retain for MSPSS scale (Velicer, 1976)

| Dimensions | Averaged Partial |
| --- | --- |
| 1 | 0.17963 |
| 2 | 0.04199* |
| 3 | 0.12825 |
| 4 | 0.58014 |

* Advised number of dimensions: 2
