# Supplementary material for: Psychometric Validation of the Multidimensional Scale of Perceived Social Support During Pregnancy in Rural Pakistan
Source: Front Psychol. 2021 Jun 15;12:601563. doi: 10.3389/fpsyg.2021.601563 (PMC8239233; doi:10.3389/fpsyg.2021.601563)
Supplement: Supplementary file 2 [file Data_Sheet_1.PDF]

F A C T O R

Unrestricted Factor Analysis

Release Version 10.3.01 x32bits

July, 2015

Rovira i Virgili University

Tarragona, SPAIN

Programming:

Urbano Lorenzo-Seva

Mathematical Specification:

Urbano Lorenzo-Seva

Pere J. Ferrando

Date: Saturday, April 24, 2021

Time: 16:39:15

DETAILS OF ANALYSIS

Participants' scores data file : D:\Work\MSPSS validation pack\Datasets\MSPSS final.dat

Method to handle missing values : Hot-Deck Multiple Imputation in Exploratory Factor Analysis (Lorenzo-Seva & Van Ginkel, 2015)

Missing code value : 999

Number of participants : 1154

Number of variables : 12

Variables included in the analysis : ALL

Variables excluded in the analysis : NONE

Number of factors : 3

Number of second order factors : 0

Procedure for determining the number of dimensions : Optimal implementation of Parallel Analysis (PA) (Timmerman, & Lorenzo-Seva, 2011)

Dispersion matrix : Pearson Correlations

Method for factor extraction : Unweighted Least Squares (ULS)

Rotation to achieve factor simplicity : Promin (Lorenzo-Seva, 1999)

Clever rotation start : Weighted Varimax

Number of random starts : 10

Maximum mumber of iterations : 100

Convergence value : 0.00001000

UNIVARIATE DESCRIPTIVES

Variable	Mean	Confidence Interval (95%)		Variance	Skewness (Zero centered)	Kurtosis
V 1	3.513	(3.42	3.60)	1.387	-0.863	-0.412
V 2	3.562	(3.48	3.65)	1.281	-0.965	-0.123
V 3	3.629	(3.55	3.71)	1.199	-1.083	0.253
V 4	3.536	(3.45	3.62)	1.308	-0.881	-0.297
V 5	3.533	(3.45	3.62)	1.197	-0.913	-0.106
V 6	3.549	(3.47	3.63)	1.201	-0.945	-0.105
V 7	3.509	(3.42	3.59)	1.236	-0.853	-0.310
V 8	3.540	(3.46	3.62)	1.231	-0.906	-0.211
V 9	2.772	(2.68	2.86)	1.439	0.222	-1.304
V 10	2.755	(2.66	2.85)	1.450	0.259	-1.242
V 11	2.863	(2.77	2.96)	1.487	0.105	-1.364
V 12	2.919	(2.83	3.01)	1.499	-0.036	-1.396

Polychoric correlation is advised when the univariate distributions of ordinal items are asymmetric or with excess of kurtosis. If both indices are lower than one in absolute value, then Pearson correlation is advised. You can read more about this subject in:

Muthén, B., & Kaplan D. (1985). A comparison of some methodologies for the factor analysis of non-normal Likert variables. British Journal of Mathematical and Statistical Psychology, 38, 171-189.

Muthén, B., & Kaplan D. (1992). A comparison of some methodologies for the factor analysis of non-normal Likert variables: A note on the size of the model. British Journal of Mathematical and Statistical Psychology, 45, 19-30.

MULTIVARIATE DESCRIPTIVES

Analysis of the Mardia's (1970) multivariate asymmetry skewness and kurtosis.

	Coefficient	Statistic	df	P
Skewness	19.463	3743.438	364	1.0000
SKewness corrected for small sample	19.463	3754.670	364	1.0000
Kurtosis	289.491	112.576		0.0000**

** Significant at 0.05

STANDARIZED VARIANCE / COVARIANCE MATRIX (PEARSON CORRELATION)

Variable	1	2	3	4	5	6	7	8	9	10	11	12
V 1	1.000											
V 2	0.735	1.000										
V 3	0.715	0.822	1.000									
V 4	0.673	0.745	0.736	1.000								
V 5	0.564	0.598	0.594	0.602	1.000							
V 6	0.572	0.594	0.604	0.611	0.896	1.000						
V 7	0.566	0.596	0.599	0.625	0.812	0.840	1.000					

V 8	0.550	0.578	0.589	0.628	0.814	0.853	0.876	1.000				
V 9	0.321	0.339	0.330	0.332	0.363	0.381	0.391	0.406	1.000			
V 10	0.319	0.347	0.328	0.308	0.350	0.350	0.364	0.379	0.873	1.000		
V 11	0.322	0.364	0.340	0.334	0.361	0.365	0.376	0.396	0.850	0.856	1.000	
V 12	0.315	0.352	0.329	0.327	0.369	0.368	0.379	0.399	0.822	0.839	0.913	1.000

ADEQUACY OF THE CORRELATION MATRIX

Determinant of the matrix = 0.000001626565253

Bartlett's statistic = 15304.0 (df = 66; P = 0.000010)

Kaiser-Meyer-Olkin (KMO) test = 0.91128 (very good)

EXPLAINED VARIANCE BASED ON EIGENVALUES

Variable	Eigenvalue	Proportion of Variance	Cumulative Proportion of Variance
1	6.94422	0.57868	0.57868
2	2.38988	0.19916	0.77784
3	1.01139	0.08428	0.86212
4	0.33200	0.02767	
5	0.28245	0.02354	
6	0.22487	0.01874	
7	0.21113	0.01759	
8	0.17601	0.01467	
9	0.12591	0.01049	

10	0.12323	0.01027
11	0.09513	0.00793
12	0.08378	0.00698

PARALLEL ANALYSIS (PA) BASED ON MINIMUM RANK FACTOR ANALYSIS

(Timmerman & Lorenzo-Seva, 2011)

Implementation details:

Correlation matrices analized: Pearson correlation matrices

Number of random correlation matrices: 500

Method to obtain random correlation matrices: Permutation of the raw data (Buja & Eyuboglu, 1992)

Variable	Real-data % of variance	Mean of random % of variance	95 percentile of random % of variance
1	61.2*	16.9	20.5
2	21.0*	15.2	18.4
3	8.3	13.6	15.7
4	2.5	12.0	13.9
5	1.9	10.5	12.2
6	1.7	9.0	10.6
7	1.0	7.5	9.4
8	0.9	6.1	8.2
9	0.8	4.5	6.5
10	0.7	3.1	5.1
11	0.2	1.6	3.4

12	0.0	0.0	0.0

* Advised number of dimensions: 2

GOODNESS OF FIT STATISTICS

Chi-Square with 33 degrees of freedom = 446.496 (P = 0.000010)

Chi-Square for independence model with 66 degrees of freedom = 15303.959

Non-Normed Fit Index (NNFI; Tucker & Lewis) = 0.95

Comparative Fit Index (CFI) = 0.97

Goodness of Fit Index (GFI) = 1.00

Adjusted Goodness of Fit Index (AGFI) = 1.00

Goodness of Fit Index without diagonal values (GFI) = 1.00

Adjusted Goodness of Fit Index without diagonal values(AGFI) = 1.00

EIGENVALUES OF THE REDUCED CORRELATION MATRIX

Variable	Eigenvalue
1	6.762529127
2	2.233731086
3	0.815667289
4	0.084521990
5	0.070568922
6	0.008806455
7	0.001815950
8	-0.012316891
9	-0.027692541

10	-0.036056438
11	-0.039746388
12	-0.049907068

UNROTATED LOADING MATRIX

Variable	F 1	F 2	F 3	Communality
V 1	0.709	-0.247	0.294	0.650
V 2	0.776	-0.269	0.405	0.838
V 3	0.763	-0.285	0.368	0.799
V 4	0.745	-0.275	0.242	0.688
V 5	0.813	-0.285	-0.289	0.825
V 6	0.834	-0.299	-0.330	0.893
V 7	0.824	-0.270	-0.283	0.832
V 8	0.830	-0.249	-0.312	0.848
V 9	0.669	0.617	-0.012	0.828
V 10	0.659	0.644	0.018	0.849
V 11	0.683	0.661	0.022	0.903
V 12	0.672	0.637	-0.000	0.857

SEMI-SPECIFIED TARGET LOADING MATRIX

Obtained from prerotation of the loading matrix

Variable	F 1	F 2	F 3
V 1	---	0.000	0.000

V 2	---	0.000	0.000
V 3	---	0.000	0.000
V 4	---	0.000	0.000
V 5	0.000	---	0.000
V 6	0.000	---	0.000
V 7	0.000	---	0.000
V 8	0.000	---	0.000
V 9	0.000	0.000	---
V 10	0.000	0.000	---
V 11	0.000	0.000	---
V 12	0.000	0.000	---

ROTATED LOADING MATRIX

Variable	F 1	F 2	F 3
V 1	0.777	0.034	0.010
V 2	0.965	-0.077	0.015
V 3	0.916	-0.025	-0.008
V 4	0.730	0.134	-0.008
V 5	0.027	0.894	-0.014
V 6	-0.016	0.966	-0.021
V 7	0.033	0.885	0.007
V 8	-0.013	0.917	0.031
V 9	-0.018	0.037	0.901
V 10	0.009	-0.021	0.927
V 11	0.016	-0.023	0.954
V 12	-0.009	0.012	0.924

ROTATED LOADING MATRIX

(loadings lower than absolute 0.300 omitted)

Variable	F 1	F 2	F 3
V 1	0.777		
V 2	0.965		
V 3	0.916		
V 4	0.730		
V 5		0.894	
V 6		0.966	
V 7		0.885	
V 8		0.917	
V 9			0.901
V 10			0.927
V 11			0.954
V 12			0.924

EXPLAINED VARIANCE AND RELIABILITY OF ROTATED FACTORS

Mislevy & Bock (1990)

Factor	Variance	Reliability estimate
1	2.948	0.930
2	3.422	0.959
3	3.441	0.963

INDICES OF FACTOR SIMPLICITY

Bentler (1977) & Lorenzo-Seva (2003)

Bentler's simplicity index (S) :   0.99995 (Percentile 100)

Loading simplicity index (LS) :   0.93987 (Percentile 100)

INTER-FACTORS CORRELATION MATRIX

Factor	F 1	F 2	F 3
F 1	1.000		
F 2	0.734	1.000	
F 3	0.412	0.437	1.000

STRUCTURE MATRIX

Variable	F 1	F 2	F 3
V 1	0.806	0.609	0.344
V 2	0.914	0.638	0.378
V 3	0.894	0.644	0.358
V 4	0.825	0.666	0.351
V 5	0.678	0.908	0.388
V 6	0.684	0.945	0.394
V 7	0.685	0.912	0.407
V 8	0.673	0.921	0.426
V 9	0.380	0.417	0.910

V 10	0.375	0.390	0.921
V 11	0.392	0.405	0.950
V 12	0.381	0.409	0.926

DISTRIBUTION OF RESIDUALS

Number of Residuals = 66

Summary Statistics for Fitted Residuals

Smallest Fitted Residual = -0.0218

Median Fitted Residual = -0.0000

Largest Fitted Residual = 0.0385

Mean Fitted Residual = 0.0000

Variance Fitted Residual = 0.0001

Root Mean Square of Residuals (RMSR) = 0.0118

Expected mean value of RMSR for an acceptable model = 0.0295 (Kelley's criterion) (Kelley, 1935,page 146; see also Harman, 1962, page 21 of the 2nd edition)

Histogram for fitted residuals

```
 Value        Freq
               |
  -0.0218       4  |  *****
  -0.0158       4  |  *****
  -0.0097       6  |  ********
```

```
-0.0037      15  |  *********************
 0.0023      27  |  ***************************************
 0.0084       5  |  *******
 0.0144       1  |  *
 0.0204       0  |
 0.0264       0  |
 0.0325       2  |  **
 0.0385       2  |  **
                +-----------+---------+---------+-----------+
                0       6.8     13.5     20.3      27.0
```

Summary Statistics for Standardized Residuals

Smallest Standardized Residual = -0.74

Median Standardized Residual = -0.00

Largest Standardized Residual = 1.31

Mean Standardized Residual = 0.00

Stemleaf Plot for Standardized Residuals

```
 -0 | 7777655544433322222111111
  0 | 0000000000000000011111111111122222236
  1 | 1223
```

DESCRIPTIVES RELATED TO MISSING DATA

Missing value code : 999

No missing data was observed in your data

References

Bentler, P.M. (1977). Factor simplicity index and transformations. Psychometrika, 59, 567-579.

Buja, A., & Eyuboglu, N. (1992). Remarks on parallel analysis. Multivariate Behavioral Research, 27(4), 509-540.

Harman, H. H. (1962). Modern Factor Analysis, 2nd Edition. University of Chicago Press, Chicago.

Kelley, T. L. (1935). Essential Traits of Mental Life, Harvard Studies in Education, vol. 26. Harvard University Press, Cambridge.

Lorenzo-Seva, U. (1999). Promin: a method for oblique factor rotation. Multivariate Behavioral Research, 34,347-356.

Lorenzo-Seva, U. (2003). A factor simplicity index. Psychometrika, 68, 49-60.

Lorenzo-Seva, U., & Van Ginkel, J. R. (2015). Multiple Imputation of missing values in exploratory factor analysis of multidimensional scales : estimating latent trait scores.Anales, in press.

McDonald, R.P. (1999). Test theory: A unified treatment. Mahwah, NJ: Lawrence Erlbaum.

Mardia, K. V. (1970), Measures of multivariate skewnees and kurtosis with applications. Biometrika, 57, 519-530.

Mislevy, R.J., & Bock, R.D. (1990). BILOG 3 Item analysis and test scoring with binary logistic models. Mooresville: Scientific Software.

Ten Berge, J.M.F., Snijders, T.A.B. & Zegers, F.E. (1981). Computational aspects of the greatest lower bound to reliability and constrained minimum trace factor analysis. Psychometrika, 46, 201-213.

Ten Berge, J.M.F., & Socan, G. (2004). The greatest lower bound to the reliability of a test and the hypothesis of unidimensionality. Psychometrika, 69, 613-625.

Timmerman, M. E., & Lorenzo-Seva, U. (2011). Dimensionality Assessment of Ordered Polytomous Items with Parallel Analysis. Psychological Methods, 16, 209-220.

Woodhouse, B. & Jackson, P.H. (1977). Lower bounds to the reliability of the total score on a test composed of nonhomogeneous items: II. A search procedure to locate the greatest lower bound. Psychometrika, 42, 579-591.

FACTOR is based on CLAPACK.

Anderson, E., Bai, Z., Bischof, C., Blackford, S., Demmel, J., Dongarra, J., Du Croz, J., Greenbaum, A., Hammarling, S., McKenney, A., & Sorensen, D. (1999). LAPACK Users' Guide. Society for Industrial and Applied Mathematics. Philadelphia, PA

FACTOR can be refered as:

Lorenzo-Seva, U., & Ferrando, P.J. (2013). FACTOR 9.2 A Comprehensive Program for Fitting Exploratory and Semiconfirmatory Factor Analysis and IRT Models. Applied Psychological Measurement, 37(6), 497-498.

Lorenzo-Seva, U., & Ferrando, P.J. (2006). FACTOR: A computer program to fit the exploratory factor analysis model.Behavioral Research Methods, Instruments and Computers, 38(1), 88-91.

For furhter information and new releases go to:

psico.fcep.urv.cat/utilitats/factor

FACTOR completed

Computing time : 0.13333333 minutes.

Matrices generated : 1002287
