# Supplementary figures and images for: Psychometric Validation of the Multidimensional Scale of Perceived Social Support During Pregnancy in Rural Pakistan
Source: Front Psychol. 2021 Jun 15;12:601563. doi: 10.3389/fpsyg.2021.601563 (PMC8239233; doi:10.3389/fpsyg.2021.601563)

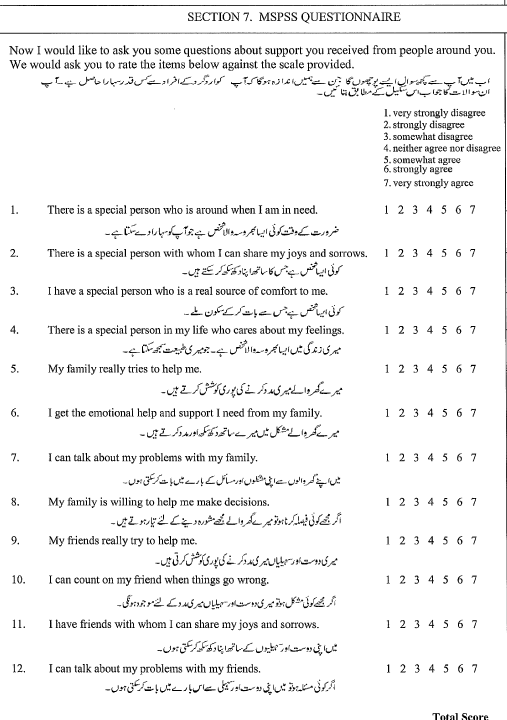

Supplement: Supplementary file 3 [file Image_1.PNG]
