# Supplementary material for: Psychometric Validation of the Multidimensional Scale of Perceived Social Support During Pregnancy in Rural Pakistan
Source: Front Psychol. 2021 Jun 15;12:601563. doi: 10.3389/fpsyg.2021.601563 (PMC8239233; doi:10.3389/fpsyg.2021.601563)
Supplement: Supplementary file 4 [file Image_2.pdf]

**Figure 2: Scree plot demonstrating number of Multidimensional scale of perceived social support factors to retain among healthy women and women with depression**

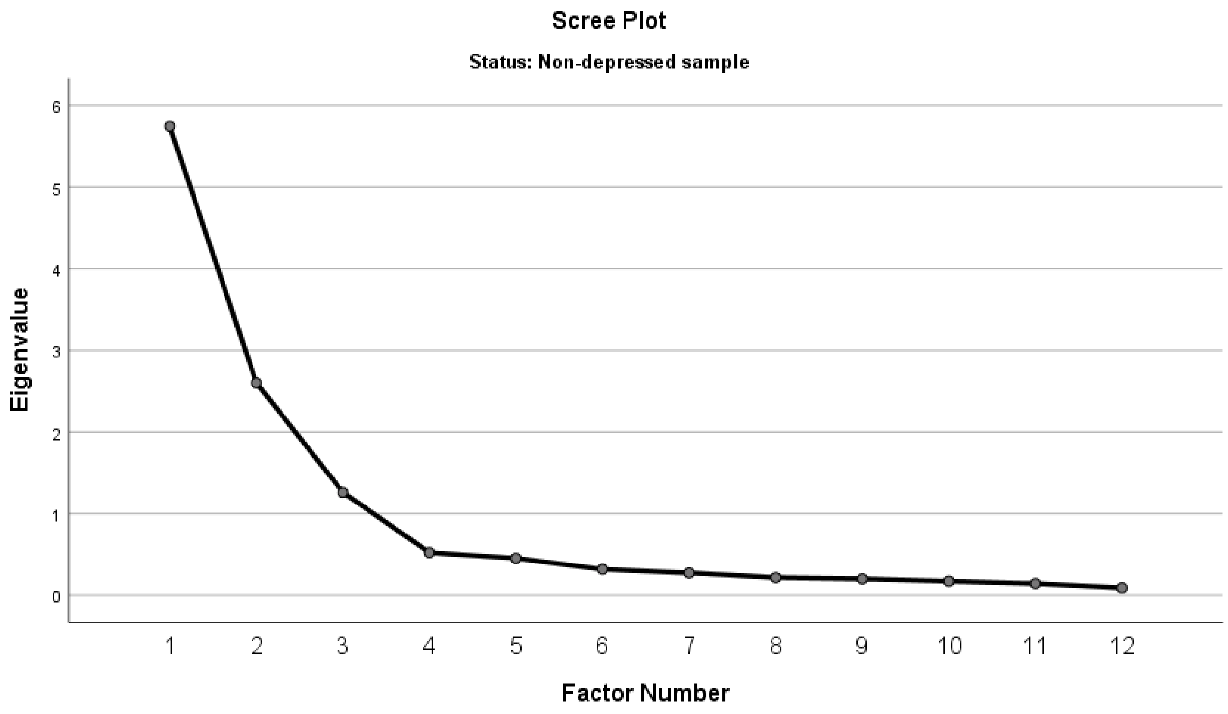

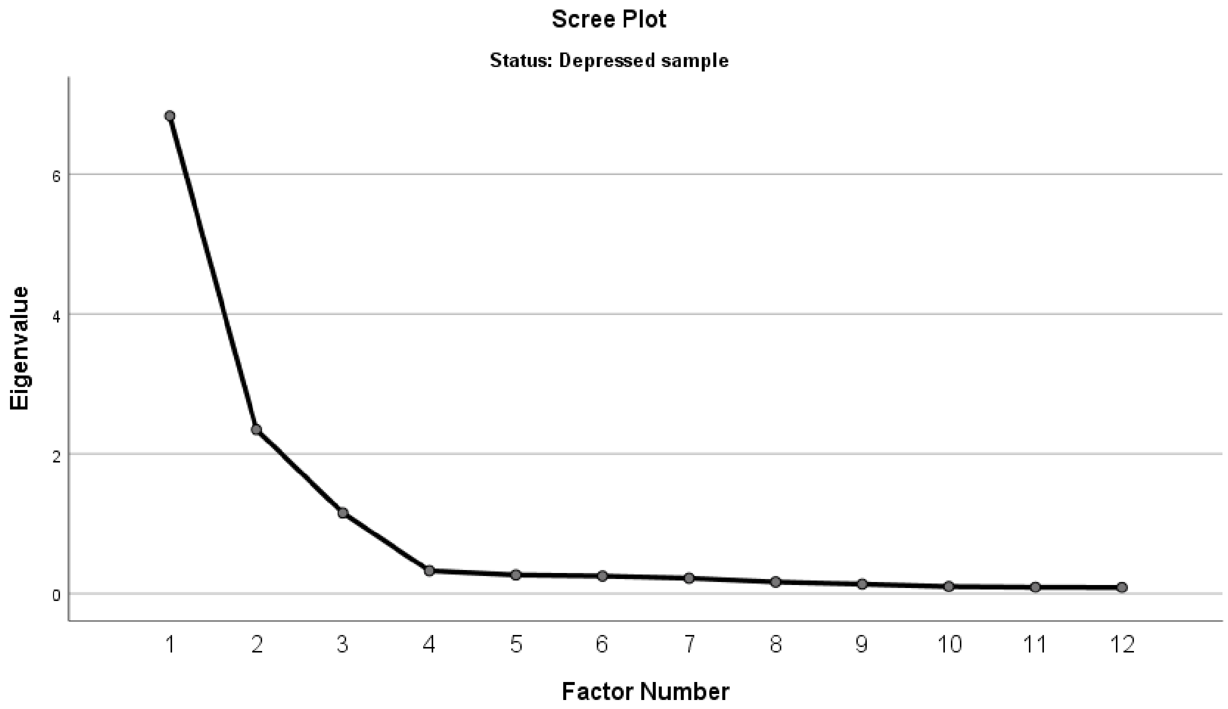
Scree Plot
Status: Depressed sample
Eigenvalue
6
4
2
0
1
2
3
4
5
6
7
8
9
10
11
12
Factor Number
